# Analysis of the survival and clinical characteristics of colorectal cancer patients with mental disorders

**DOI:** 10.1002/ags3.12421

**Published:** 2021-01-25

**Authors:** Junji Kurashige, Masaaki Iwatsuki, Kosuke Mima, Daichi Nomoto, Hironobu Shigaki, Kohei Yamashita, Takeshi Morinaga, Shiro Iwagami, Nobutomo Miyanari, Hideo Baba

**Affiliations:** ^1^ Department of Surgery National Hospital Organization Kumamoto Medical Center Kumamoto Japan; ^2^ Department of Gastroenterological Surgery Graduate School of Medical Sciences Kumamoto University Kumamoto Japan

**Keywords:** CRC, mental disorder

## Abstract

**Aim:**

Many studies have shown that patients with mental disorders are less likely than non‐psychiatric patients to be diagnosed with or treated for various types of cancers because of their low awareness and understanding of the disease as well as reduced ability to cooperate with medical staff. We analyzed the clinical features of patients with colorectal cancer (CRC) and preexisting mental illness.

**Methods:**

All patients underwent primary tumor resection for CRC. We reviewed the records of 68 patients who were diagnosed with mental disorders. The patients' clinicopathological information was compared with that of a control group of 893 CRC patients.

**Results:**

There was no significant difference in the overall disease stage at the time of surgery between the groups. However, disease‐free survival, cancer‐specific survival, and overall survival were significantly worse in the mental disorder group than in the control group (*P* < .01). In particular, among those with stage III CRC, overall survival was significantly worse in the patients with mental disorders than in the non‐psychiatric patients (*P* < .001). The frequency of complications of ≥grade 2 according to the Clavien‐Dindo classification was higher in the SMI group because of postoperative paralytic ileus.

**Conclusions:**

Advanced CRC patients with mental disorders are less likely to receive postoperative adjuvant chemotherapy or treatment for recurrent cancer than CRC patients without mental disorders; therefore, they experience worse outcomes. Collaboration across multiple departments is necessary for managing CRC patients with mental disorders.

## Graphical abstract

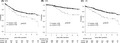

## 1 INTRODUCTION

Patients with mental disorders, such as schizophrenia, bipolar disorder, schizoaffective disorder, and some depressive disorders, exhibit high rates of all‐cause morbidity and mortality, and many studies have found that such patients are at increased risk of metabolic syndrome and cardiovascular diseases.^1^, ^2^, ^3^, ^4^, ^5^, ^6^ Several studies have indicated that the incidence of cancer is not higher among patients with mental disorders than among the general population.^7^, ^8^, ^9^, ^10^, ^11^ Other studies have indicated that for specific types of cancers, including breast cancer,^12^, ^13^, ^14^ pancreatic carcinoma,^15^ prostate cancer,^16^ and lung cancer,^17^ patients with mental disorders exhibit worse mortality rates than non‐psychiatric patients.^18^ It has been reported that the reasons for the high mortality among cancer patients with mental disorders are reduced use of surgery, radiotherapy, and chemotherapy.^17^

Colorectal cancer (CRC) is the third most common type of cancer in the world,^19^ and the incidence of CRC has been increasing in recent years in Japan.^20^ While the link between mental disorder and cancer survival is well‐established, few studies have specifically examined this issue with regard to CRC. Several nationwide cohort studies have reported CRC in patients with psychiatric disorders. In the United States, Baillargeon et al examined CRC among the elderly population and reported that people with preexisting mental disorders were less likely to be diagnosed with local cancer and more likely to be diagnosed with cancer of unknown stage, not undergo surgery, radiotherapy, or chemotherapy, and have a higher cancer‐specific mortality risk than those without a history of mental disorders.^21^ In New Zealand, Cunningham et al found that patients with a history of use of psychiatric services exhibited worse survival after being diagnosed with CRC than those who did not have such a history.^12^ In Finland, Manderbacka et al reported that CRC patients with psychotic or substance misuse disorders displayed higher cancer‐specific mortality than other patients with CRC, which was only partly explained by differences in the stage at presentation and the treatments administered.^22^

However, few studies have reported the details of treatment for CRC patients with mental disorders and examined whether the outcomes of CRC vary between patients with and without a history of mental disorders.

The aim of the current study was to examine the independent and aggregate effects of a preexisting diagnosis of any mental disorder on cancer stage at diagnosis, the details of treatment administered, and survival of CRC patients.

## 2 METHODS

### 2.1 Patients

We enrolled a total of 961 CRC (any stage) patients who underwent primary tumor resection at National Hospital Organization Kumamoto Medical Center, Kumamoto, Japan, between January 2008 and March 2017. This hospital is the only designated emergency general hospital with a psychiatric ward in the prefecture. Patients with mental disorders were defined as patients who were diagnosed by an attending psychiatrist and needed to be admitted to a psychiatric ward for difficulties in living independently, according to a previous study.^23^ All patients with psychiatric disorders had been diagnosed with psychiatric disorders before CRC. No information regarding the severity or subclassification of each psychiatric disorder was available. The clinicopathological data of the mental disorder group were compared with those of non‐psychiatric patients (the control group). Adjuvant therapy was selected for each patient based on the American Society of Clinical Oncology (ASCO)^24^, ^25^ and the European Society of Medical Oncology (ESMO) guidelines.^26^ The TNM classification (seventh edition) was used for staging. This study was approved by the institutional review board of the National Hospital Organization Kumamoto Medical Center and all patients provided written informed consent.

### 2.2 Statistical analysis

Inter‐group differences in clinicopathological factors were evaluated using analysis of variance for continuous variables and Pearson's chi‐squared test for categorical variables. Disease‐free survival (DFS), cancer‐specific survival (CSS), and overall survival (OS) were assessed from the time of the first surgery until the date of death or the last follow‐up. DFS was defined as the time between the day of surgery and the day of recurrent disease. Post‐recurrence survival was defined as the time between the first recurrence and death or the last follow‐up. Survival curves were constructed using the Kaplan‐Meier method, and the statistical significance of differences between the two groups was assessed using the log‐rank test. For all analyses, the Statistical Package for the Social Sciences and the software R (version 3.3.1) were used. *P*‐values of <.05 were considered statistically significant.

## 3 RESULTS

### 3.1 Patient characteristics

Of the 961 patients with CRC, 68 had been diagnosed with mental disorders (mental disorder group) (Table 1). The mental disorders were schizophrenia (n = 33); dementia, including Alzheimer's disease, senile dementia, and dementia with Lewy bodies (n = 24); intellectual disabilities (n = 8); and manic depression (n = 3). The control group (n = 893) also had patients with psychiatric disorders (n = 130), such as schizophrenia (n = 21), dementia (n = 24), manic depression (n = 3), and others (n = 38), but because their symptoms were mild and could be managed in general wards, they were not included in the mental disorder group. On the day of surgery for cancer, the CRC patients with mental disorders had significantly lower mean body mass indices and a significantly higher incidence of rectal cancer than the control group. There were no significant differences in the median age at the time of surgery and sex distribution between the groups (Table 1).

**TABLE 1:** Background, tumor, and treatment characteristics

Features	Overall	Control group		SMI group		*P‐*value
	n = 961, %	n = 893, %		n = 68, %		
Age (years)						
Median (Range)	74.0, (30‐101)	74.0 ( 30‐101)		71.0 ( 34‐93)		.090
Gender						
Male	492, 51.2%	454	50.8%	38	55.9%	.452
Female	469, 48.8%	439	49.2%	30	44.1%	
BMI						
Mean ± SD	22.0 ± 3.8	22.0 ± 3.9		21.0 ± 3.2		
Tumor site						
Colon	675, 70.2%	637	71.3%	38	55.9%	
Rectum	286, 29.8%	256	28.7%	30	44.1%	
Distant metastasis						
Present	111, 11.6%	101	11.3%	10	14.7%	.306
Absent	850, 88.4%	792	88.7%	58	85.3%	
Emergency operation						
Emergency	62, 6.5%	56	6.3%	6	8.8%	.437
Elective	899, 93.5%	837	93.7%	62	91.2%	
Surgical approach						
Open	595, 61.9%	547	61.3%	48	70.6%	.154
Laparoscopic	366, 38.1%	346	38.7%	20	29.4%	
Operation time (min)						
Mean ± SD	269.7 ± 128.9	268.8 ± 129.4		282.4 ± 124.4		.402
Intraoperative bleeding (ml)						
Mean ± SD	295.3 ± 553.4	285.6 ± 540.9		424.3 ± 695.7		.064
Tumor depth						
pT0,1,2	200, 20.8%	186	20.8%	14	20.6%	1.000
pT3,4	761, 79.2%	707	79.2%	54	79.4%	
Lymph node metastasis						
Present	393, 40.9%	363	40.6%	30	44.1%	.610
Absent	568, 59.1%	530	59.4%	38	55.9%	
Pathological stage						
0/I	170, 17.7%	157	17.6%	13	19.1%	.664
II	367, 38.2%	345	38.6%	22	32.4%	
III	313, 32.6%	290	32.5%	23	33.8%	
IV	111, 11.6%	101	11.3%	10	14.7%	
Complication						
≥Grade 2 CDC complications	165, 17.2%	144	16.1%	21	30.9%	.004*
≥Grade 3 CDC complications	97, 10.1%	88	9.9%	9	13.2%	.401

* *P*‐value < .05

### 3.2 Treatment, cancer characteristics, and morbidity

Table 1 shows that the number of emergency operations, surgical approach, operation time, amount of intraoperative bleeding, tumor depth, frequency of lymph node metastasis, and pathological stage did not differ significantly between the groups. However, the frequency of postoperative complications of ≥grade 2 according to the Clavien‐Dindo classification was higher in the mental disorder group than in the control group. Postoperative paralytic ileus (a grade 2 complication) was more common in the mental disorder group (4/68 = 6.0%) than in the control group (14/893 = 1.6%, *P* = .035). Therefore, the frequency of ≥grade 2 complications was high in the mental disorder group. There was no difference in the frequency of complications of ≥grade 3 according to the Clavien‐Dindo classification between the groups.

### 3.3 Survival outcomes of patients with CRC who underwent radical resection

**FIGURE 1:**
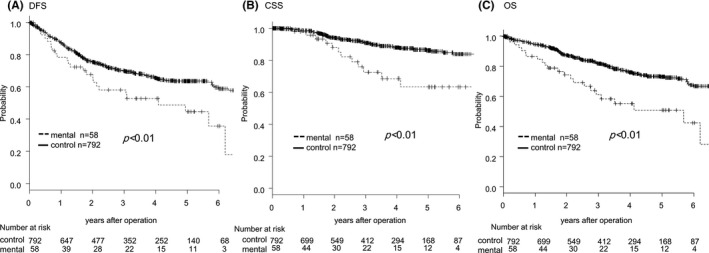
Kaplan‐Meier survival curves of CRC patients with and without mental disorders who underwent radial resection. (A) Disease‐free survival curves: the data for patients with (dashed line) and without (solid line) mental disorders were compared, (B) Cancer‐specific survival curves, (C) Overall survival curve

Figure 1 shows the DFS, CSS, OS, of CRC patients who underwent radical resection. Patients with mental disorders exhibited significantly worse prognoses in terms of DFS, CSS, and OS than the patients in the control group. Supplement Figure S1 shows OS curve for CRC patients with control, dementia, and other mental illness (eg, schizophrenia, intellectual disabilities). There is no significant difference in overall survival between dementia and other.

**FIGURE 2:**
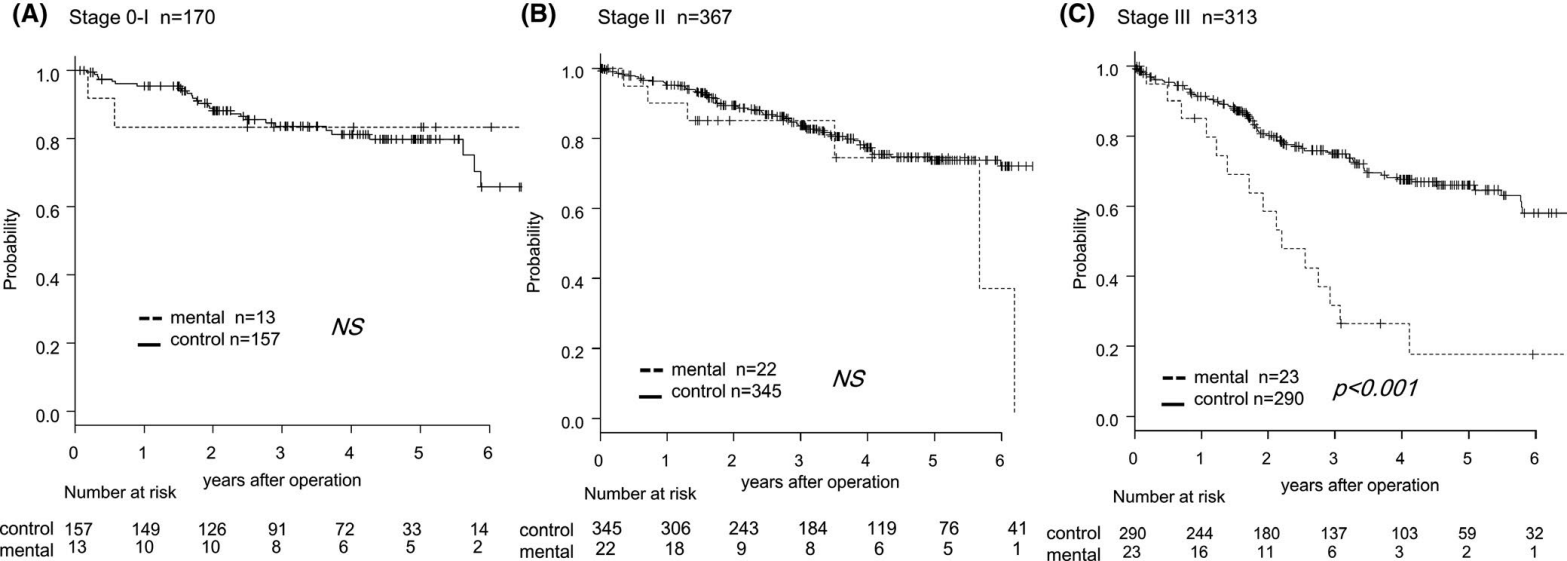
Overall survival curves of CRC patients with and without mental disorder. The data for patients with (dashed line) and without (solid line) mental disorders were compared. (A) Stage0‐I, (B) Stage II, (C) Stage III

In particular, regarding stage III CRC, the patients with mental disorders exhibited significantly worse OS than the patients in the control group (Figure 2). Among the patients with stage III CRC, no significant differences in age at the time of surgery, sex distribution, mean BMI, surgical approach, operation time, and amount of intraoperative bleeding were observed between the groups. Compared with the control group, few patients in the mental disorder group with stage III CRC received postoperative adjuvant chemotherapy after curative surgery (Table 2).

**TABLE 2:** Background, tumor, and treatment characteristics of the stage III patients

Features	Overall	Control group		SMI group		*P‐*value
	n = 313, %	n = 290, %		n = 23, %		
Age (years)						
Median (range)	74.0 (30‐101)	74.0 (30‐101)		73.0 ( 34‐93)		.478
Gender						
Male	157, 50.2%	144	49.7%	13	56.5%	.666
Female	156, 49.8%	146	50.3%	10	43.5%	
BMI						
Mean ± SD	21.9 ± 3.9	21.9 ± 4.0		21.11 ± 3.1		.378
Tumor site						
Colon	209, 66.8%	198	68.3%	11	47.8%	.064
Rectum	104, 33.2%	92	31.7%	12	52.2%	
Surgical approach						
Open	196, 62.6%	180	62.1%	16	69.6%	.194
Laparoscopic	117, 37.4%	110	37.9%	7	30.4%	
Operation time (min)						
Mean ± SD	269.7 ± 128.9	276.5 ± 139.4		311.4 ± 147.4		.250
Intraoperative bleeding (g)						
mean ± SD	295.3 ± 553.4	346.3 ± 683.2		438.7 ± 598.3		.645
Tumor depth						
pT0,1,2	27, 8.6%	26	9.0%	1	4.3%	.706
pT3,4	286, 91.4%	264	91.0%	22	95.7%	
Adjuvant chemotherapy						
Yes	155, 49.5%	152	52.4%	3	13.0%	<.01*
No	158, 50.5%	138	47.6%	20	87.0%	

* *P*‐value < .05

### 3.4 Survival outcomes of CRC patients after recurrence

**FIGURE 3:**
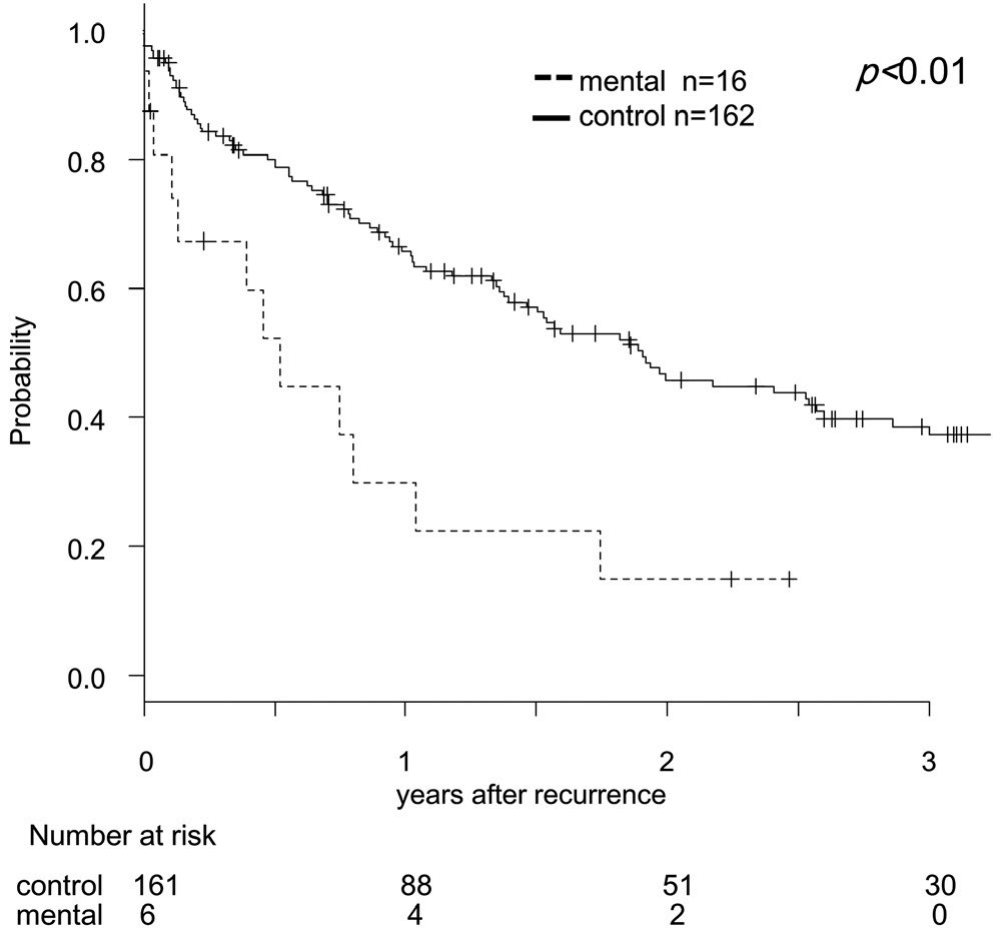
Post‐recurrence survival curve of CRC patients with and without mental disorders. The data for patients with (dashed line) and without (solid line) mental disorders were compared

In our study, 178 of the 850 CRC patients who underwent radical resection had recurrent cancer, including 16/58 (22.4%) patients with mental disorders and 162/792 (20.5%) control patients. Only one of the patients with mental disorders who developed recurrent cancer underwent surgical treatment, whereas the other 15 patients with mental disorders who developed recurrence received supportive care. On the other hand, 109/162 (67.3%) control patients with recurrent cancer received treatment, such as chemotherapy, radiotherapy, or surgery. Figure 3 shows the post‐recurrence survival of patients with recurrence. Patients with mental disorders exhibited significantly worse prognoses in terms of post‐recurrence survival than patients in the control group (*P* < .01).

## 4 DISCUSSION

We retrospectively analyzed the clinicopathological and survival data of patients with CRC (any stage) who underwent primary surgery and compared the outcomes of patients with and without mental disorders. We found that there was no significant difference in the overall stage at the time of surgery between the groups, but the frequency of postoperative complications of ≥grade 2 according to the Clavien‐Dindo classification was higher in the mental disorder group. Among the patients with stage III CRC who underwent curative resection, fewer patients with mental disorders underwent postoperative adjuvant chemotherapy, and OS was significantly worse in the mental disorder group than in the control group. Moreover, in patients who developed recurrence, fewer patients with mental disorders underwent several treatments than the control group, and post‐recurrence survival was significantly worse in the mental disorder group than in the control group.

Patients with mental disorders are often less aware of their disease, exhibit less understanding of their disease, and are less likely to cooperate during their care than non‐psychiatric patients, posing a difficulty for appropriate diagnosis and treatment of several types of cancers. In patients with mental disorders, tumors are often advanced at the time of diagnosis.^8^, ^27^, ^28^ However, in the present study, we found that the overall stage of CRC at the time of surgery did not differ significantly between the groups. This was probably because in Japan they had been admitted to psychiatric facilities that had discovered their cancer symptoms (bleeding or abdominal pain) or they had undergone frequent screening examinations. Therefore, the rate of rectal cancer was high in the mental disorder group. One of the limitations of this study was its biased cohort; that is, this study did not include patients who did not undergo primary surgery. This study only evaluated the clinical features of surgical cases.

The frequency of complications of ≥grade 2 according to the Clavien‐Dindo classification was higher in the mental disorder group than in the control group; however, the frequency of ≥grade 3 complications (including mortality) did not differ significantly between the groups. The frequencies of anastomotic leakage and intra‐abdominal abscess formation (≥grade 3 complications) did not differ significantly between the groups, whereas postoperative paralytic ileus (a grade 2 complication) was more common in the mental disorder group than in the control group. Therefore, the frequency of ≥grade 2 complications was higher in the mental disorder group. Most patients with mental disorders were taking antipsychotics and were inactive, which was likely to lead to paralytic postoperative ileus.

In the clinical setting, the medical staff might have difficulty selecting treatment plans for patients with mental disorders because of problems associated with obtaining informed consent from the patients themselves or the medical insurance system. In our analysis, some treatment‐related factors were shown to differ among the groups. We recommend adjuvant chemotherapy for stage III CRC and high‐risk stage II CRC based on the ASCO and ESMO guidelines. Among the patients with stage III CRC who underwent curative resection, the percentage of patients who received postoperative adjuvant chemotherapy was lower in the mental disorder group than in the control group (13.0% vs 52.4%, Table 2). This might explain the differences in the recurrence rate and DFS observed between the groups. Similarly, among the patients with high‐risk stage II CRC who underwent curative resection, the percentage of patients who received postoperative adjuvant chemotherapy was lower in the mental disorder group than in the control group (0%, 0/17% vs 32.6%, 93/285). In our study, 178 of the 850 CRC patients who underwent radical resection developed recurrent cancer. Only one of the patients with mental disorders who developed recurrent cancer underwent surgical treatment, whereas the other 15 patients with mental disorders received best supportive care. On the other hand, 109/162 (67.3%) control patients with recurrent cancer received treatment, such as chemotherapy, radiotherapy, or surgery. It is worth noting that few patients with mental disorders received adjuvant chemotherapy or treatment after recurrence, owing to the difficulty in obtaining informed consent. The patients with SMI may have a limited understanding of the clinical course of their cancer and the effects of mental illness (e.g. reduced motivation, cognitive impairment, and active psychosis) make it much more difficult to undergo complex cancer regimens. In addition, psychiatric patients often have limited access to treatment services for physical complaints.

If patients with mental disorders are unable to comply with treatment, careful liaison and effective communication with psychiatrists, particularly psycho‐oncologists (where available), can be very helpful. In order to provide chemotherapy to patients with mental disorders, cooperation across multiple departments, including medical oncologists, surgeons, and psycho‐oncologists, is indispensable.^29^ However, several studies have reported that physicians may avoid choosing invasive treatments for advanced cancer patients with psychiatric conditions.^8^, ^18^, ^27^, ^28^ In addition, there are no specific guidelines on the best supportive care for cancer patients with psychiatric disorders, and there are very few empirical studies on palliative care for cancer patients with mental disorders.^30^ It can be difficult to administer standard treatments to patients with mental disorders; therefore, a limited therapeutic plan is sometimes adopted. Due to aging populations, the number of patients with mental disorders, especially dementia, is increasing worldwide. As the global population ages further, the number of patients with dementia is estimated to double to 81.1 million by the year 2040.^31^ Thus, the clinical management of patients with CRC and mental illness will become more important.

The limitations of this study include the small number of subjects and the biased nature of the cohort. We analyzed the cases of 961 patients over a 10‐year period in a single‐center retrospective study. In addition, we only analyzed patients who underwent primary surgical treatment. To clarify the overall status of CRC patients with mental disorders, patients with non‐resected cancer must also be analyzed. Furthermore, the control group included patients with mild mental illness. In fact, there were many patients with senile dementia in the control group who were not admitted to a psychiatric ward. It is possible that these patients did not receive appropriate treatment because of their psychiatric symptoms.

In conclusion, CRC patients with mental disorders underwent primary resection without severe complications. In addition, CRC patients with mental disorders received postoperative adjuvant chemotherapy and treatment for recurrence less frequently than the control patients. Therefore, the outcomes of CRC patients with mental disorders might be worse than those of CRC patients without mental disorders.

## CONFLICT OF INTEREST

Authors declare no conflict of interests for this article.

## Supporting information

Fig S1

Supplementary Material
