# Supplementary figures and images for: Analysis of the survival and clinical characteristics of colorectal cancer patients with mental disorders
Source: Ann Gastroenterol Surg. 2021 Jan 25;5(3):314–20. doi: 10.1002/ags3.12421 (PMC8164452; doi:10.1002/ags3.12421)

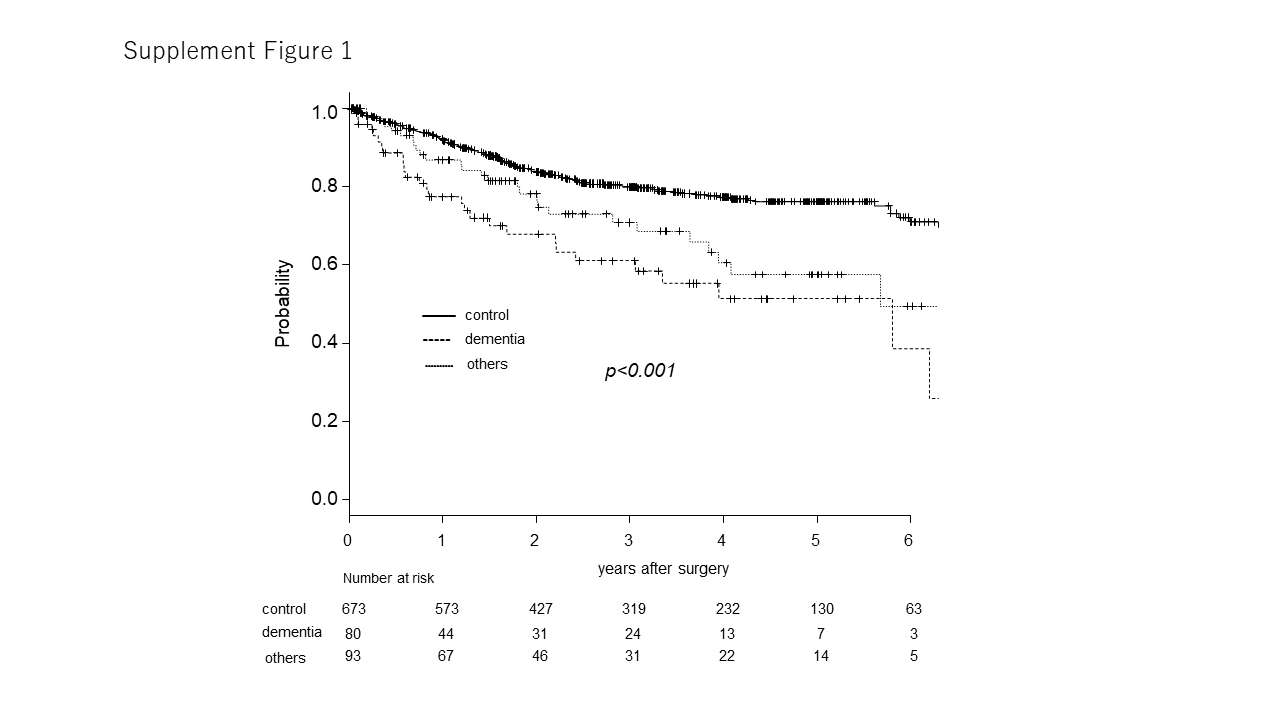

Supplement: Supplementary file 1 — Fig S1 [file AGS3-5-314-s002.tif]
