# Supplementary material for: Analysis of the survival and clinical characteristics of colorectal cancer patients with mental disorders
Source: Ann Gastroenterol Surg. 2021 Jan 25;5(3):314–20. doi: 10.1002/ags3.12421 (PMC8164452; doi:10.1002/ags3.12421)
Supplement: Supplementary file 2 — Supplementary Material [file AGS3-5-314-s001.docx]

Supplement Figure 1.

Overall survival curves of CRC patients with dementia, other mental illness or control. The data for patients with dementia (dashed line), other mental illness (dotted line), control (solid line) was compared.
